# A Mixture of Atropisomers Enhances Neutral Lipid Degradation in Mammalian Cells with Autophagy Induction

**DOI:** 10.1038/s41598-018-30679-0

**Published:** 2018-08-14

**Authors:** Keisuke Kobayashi, Satoshi Ohte, Taichi Ohshiro, Narihiro Ugaki, Hiroshi Tomoda

**Affiliations:** Graduate School of Pharmaceutical Sciences, Kitasato University, 5-9-1 Shirokane, Minato-ku, Tokyo 108-8641 Japan

## Abstract

Atropisomers with a biaryl dihydronaphthopyranone structure, dinapinones A1 (DPA1) (*M* position) and A2 (DPA2) (*P* position), were isolated from the fungus culture broth of *Talaromyces pinophilus* FKI-3864 as inhibitors of [^14^C]neutral lipid ([^14^C]triacylglycerol (TG) and [^14^C]cholesteryl ester (CE)) synthesis from [^14^C]oleic acid in Chinese hamster ovary-K1 (CHO-K1) cells. DPA2 inhibited [^14^C]TG and [^14^C]CE synthesis (IC_50_s_,_ 0.65 and 5.6 μM, respectively), but DPA1 had no inhibitory activity on [^14^C]TG and [^14^C]CE synthesis even at 12 μM. However, a 1:1 mixture of DPA1 and DPA2 (DPA_mix_) had the most potent inhibitory activity on [^14^C]TG and [^14^C]CE synthesis (IC_50_s, 0.054 and 0.18 μM, respectively). The mechanism of action of DPA_mix_ was investigated. DPA_mix_ had no effects on the enzymes involved in neutral lipid synthesis, while DPA_mix_ enhanced the degradation of [^14^C]neutral lipids with concomitant decrease in cytosolic lipid droplets accumulated in CHO-K1 cells. From analysis of autophagy marker proteins, DPA_mix_ caused dose-dependent induction of microtubule-associated protein light chain 3-II (LC3-II) and degradation of p62. In the autophagic flux assay using bafilomycin A_1_, DPA_mix_ upregulated autophagosome turnover. These results reveal that DPA_mix_ enhances neutral lipid degradation together with induction of autophagy.

## Introduction

Neutral lipids, including triacylglycerol (TG) and cholesteryl ester (CE), are the final storage forms of free long-chain fatty acids and cholesterol in mammals; these molecules are used for vital functions, such as membrane function, epidermal integrity, bile acid synthesis, lipoprotein trafficking and steroidogenesis^1^. However, excess cytoplasmic accumulation of neutral lipids is a significant risk factor for several disease pathologies, including diabetes, obesity, atherosclerosis and nonalcoholic fatty liver disease^2^. For example, the accumulation of CE causes macrophages in vessels to convert to foam cells, resulting in the development of atherosclerotic plaques and subsequent myocardial and cerebral infarction^3^. Similarly, the accumulation of TG in adipocytes can lead to obesity and thus contributes to fat formation in all obesity syndromes^4^. We are interested in discovering microbial inhibitors of neutral lipid synthesis/accumulation using a variety of cell-based assays with mouse peritoneal macrophages^5–12^, Raji cells^13^, and CHO cells expressing sterol *O*-acyltransferase (SOAT, also known as acyl-CoA: cholesterol acyltransferase (ACAT))^14–18^, among others.

**Figure 1:**
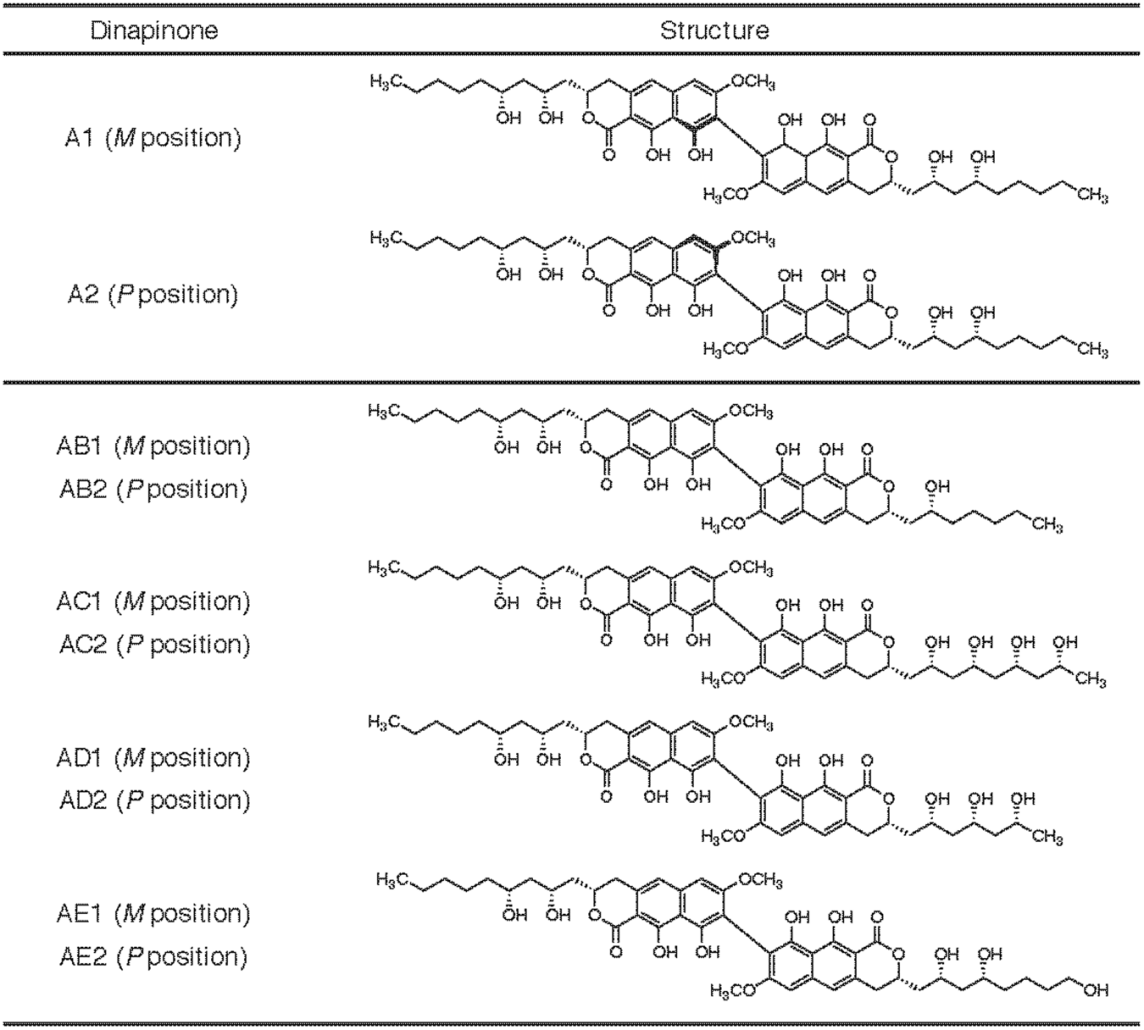
Structures of dinapinones. Dinapinones A1 and A2 are homodimers, and the others are heterodimers.

In our recent screening program, dinapinone (DP) A and congeners were isolated from the culture broth of *Talaromyces pinophilus* FKI-3864^19–21^ (Fig. 1). DPA showed very potent inhibition of the synthesis of [^14^C]neutral lipids [^14^C]TG and [^14^C]CE from [1-^14^C]oleic acid in CHO-K1 cells (IC_50_ values, 0.097 and 0.31 μM, respectively). Initially, DPA was considered a single compound, but DPA consists of the atropisomers dinapinone A1 (DPA1) (*M* position) and A2 (DPA2) (*P* position) at a ratio of 2:3 (Fig. 1). Unfortunately, DPA2 inhibited [^14^C]TG and [^14^C]CE synthesis with higher IC_50_ values (0.65 and 5.6 μM, respectively) in CHO-K1 cells, and DPA1 showed no inhibitory effects (IC_50_: >12 μM). Interestingly, when purified DPA1 and DPA2 were mixed at various ratios to evaluate their activity in cells, a 1:1 mixture (DPA_mix_) exhibited the most potent inhibitory activity on [^14^C]TG synthesis, with an IC_50_ of 0.054 μM. As shown in the present study, a combination of DP atropisomers (*M* and *P* position) is essential for inhibitory activity, and DPs do not affect the neutral lipid biosynthesis pathway but do enhance neutral lipid degradation along with inducing autophagy.

## Results

### Effects of DPA_mix_ on neutral lipid synthesis in mammalian cells

**Figure 2:**
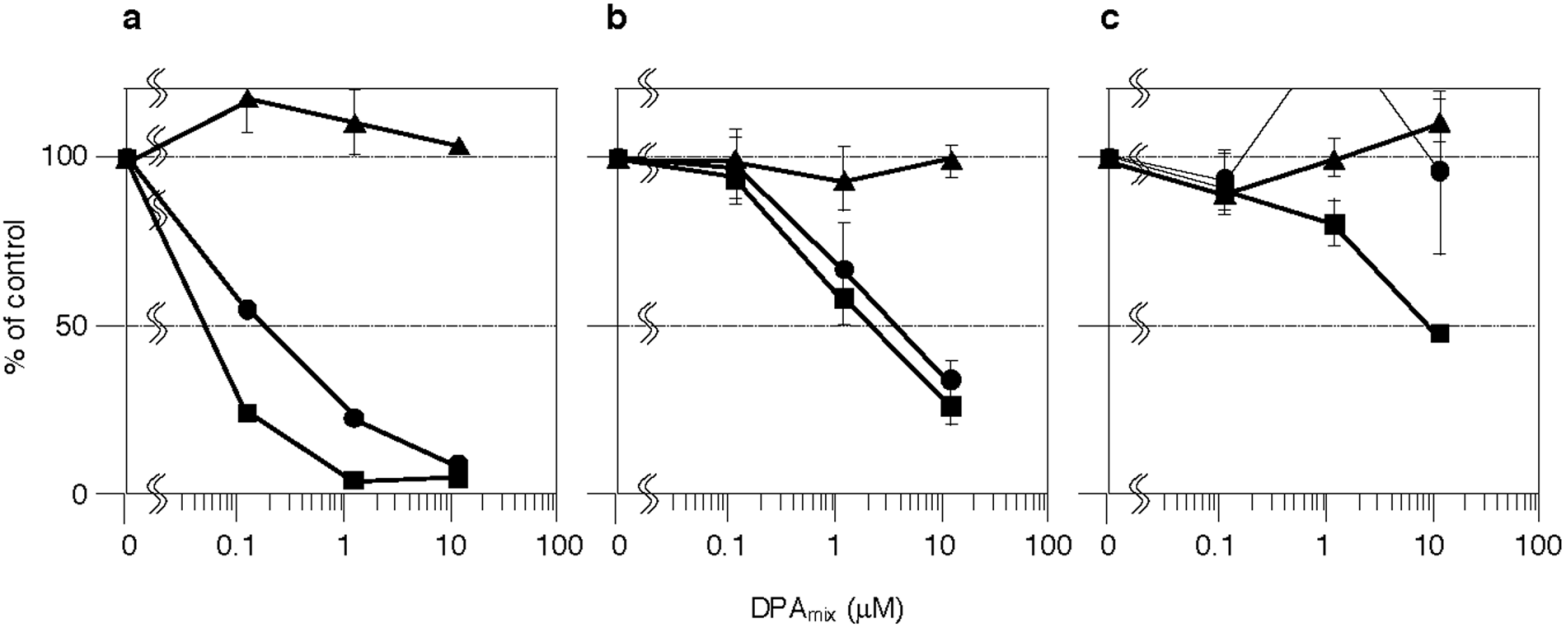
Effects of DPA_mix_ on neutral lipid accumulation in three mammalian cells. After CHO-K1 cells (**a**), HeLa S3 cells (**b**) and HepG2 cells (**c**) were cultured with DPA_mix_ and [^14^C]oleic acid for 6 h, the cells were lysed and [^14^C]TG (■), [^14^C]CE (●) and [^14^C]PL (▲) were quantified by an image analyzer. The results obtained were plotted as % of control (without drugs). Values represent means ± SD (n = 3 ~ 4).

Consistent with a previous report^19^, DPA_mix_ inhibited [^14^C]TG synthesis from [^14^C]oleic acid with IC_50_ values of 0.054 and 0.090 μM in CHO-K1 cells and Raji cells (only TG synthesis was observed^22^), respectively. First, we examined the effects of the mixture of DPA1 and DPA2 at various ratios on CE and TG synthesis in CHO-K1 cells. As shown in Table 1, mixtures of 1:1 to 1:5 (DPA1 ≤ DPA2) potently inhibited CE synthesis, but DPA1 alone and mixtures of 5:1 to 2:1 DPA1 and DPA2 (DPA1 > DPA2) did not inhibit CE synthesis. Interestingly, 1:1 to 1:3 mixtures exhibited the most potent CE inhibitory activity, with IC_50_ values of 0.18–0.19 μM. This tendency for DPA mixtures to inhibit CE synthesis was similar to the inhibition of TG synthesis. This finding prompted us to investigate the effects of a 1:1 mixture of DPA1 and DPA2 (DPA_mix_) on neutral lipid synthesis in other mammalian cells. In HeLa S3 cells, DPA_mix_ inhibited [^14^C]TG and [^14^C]CE synthesis (IC_50_: 2.3 and 4.0 μM, respectively) in a dose-dependent manner and, when compared to CE inhibition, showed rather selective TG inhibition, similar to CHO-K1 cells (Fig. 2a,b). In HepG2 cells, DPA_mix_ inhibited [^14^C]TG synthesis in a dose-dependent manner (IC_50_: 9.5 μM) (Fig. 2c) but exhibited no effects on [^14^C]CE synthesis. This difference might arise from the fact that HepG2 cells exhibit weak CE synthesis activity and produce very low levels of [^14^C]CE (approximately one-fifteenth of the level of [^14^C]TG), and thus these cells probably do not provide reliable CE data. The cytotoxicity of DPA_mix_ on all the cell lines tested above was tested by the MTT assay. As shown in Supplemental Fig. 1, DPA_mix_ did not show cytotoxicity, even after treatment with 12 μM for 6 h. Based on these findings, DPA_mix_ affected neutral lipid synthesis and was more active toward TG synthesis than CE synthesis in mammalian cell lines. Further experiments were performed using CHO-K1 cells.

**Table 1:** Effect of a mixture of DPA1 and DPA2 at a different ratio on neutral lipid synthesis in CHO-K1 cells.

Ratio (A1: A2)	IC_50_ (μM)	
	TG	CE
1: 0	>12	>12
5: 1	>1.2	>1.2
4: 1	>1.2	>1.2
3: 1	>1.2	>1.2
2: 1	>1.2	>1.2
1: 1	0.054	0.18
1: 2	0.073	0.19
1: 3	0.16	0.18
1: 4	0.25	0.71
1: 5	0.24	0.51
0: 1	0.65	>12

### Effects of other atropisomers of DPs on neutral lipid synthesis in CHO-K1 cells

Since a 1:1 mixture of DPA1 and DPA2 completely inhibited neutral lipid synthesis, we next investigated whether other DPs exerted the same effect. Heterodimers of DPAB, DPAC, DPAD and DPAE consist of the atropisomers DPAB1, DPAC1, DPAD1, DPAE1 (1 series with the *M* position) DPAB2, DPAC2, DPAD2 and DPAE2 (2 series with the *P* position), respectively^21^ (Fig. 1). As shown in Table 2, with the exception of DPAE1, the administration of the 1 series of DP alone did not show inhibitory effects, even at 11 - 12 μM, whereas the administration of the 2 series of DP alone, with the exception of DPAC2, inhibited [^14^C]TG synthesis (IC_50_: 1.1–4.3 μM). These results were consistent with the outcomes for DPA. Interestingly, DPAE1 moderately inhibited [^14^C]TG synthesis (IC_50_: 4.8 μM), whereas DPAC2 showed no inhibitory activity. The discrepancy might be due to effects of different alkyl side chains at the C-3′ in the heterodimer form of DP on inhibitory activity. Furthermore, except for DPAC_mix_, 1:1 mixtures of the atropisomers of heterodimeric DPs (DPAB_mix_, DPAD_mix_ and DPAE_mix_) exhibited enhanced inhibitory activity on [^14^C]TG and [^14^C]CE synthesis in CHO-K1 cells, similar to DPA_mix_ (Table 2). Among the heterodimeric DPs, DPAB_mix_ exhibited the strongest inhibition of TG (IC_50_: 1.1 to 0.030 μM, 37-fold) and CE (IC_50_: >12 to 0.089 μM, >135-fold) synthesis. Thus, both atropisomers (*M* and *P* position) of a DP are essential for complete inhibition of neutral lipid synthesis in CHO-K1 cells. Therefore, subsequent studies of the mechanism of action were performed using DPA_mix_.

**Table 2:** Effect of dinapinones on neutral lipid synthesis in CHO-K1 cells.

Compound	IC_50_ (μM)	
	TG	CE
DPA1	>12	>12
DPA2	0.65	>12
DPA_mix_	0.054	0.18
DPAB1	>12	>12
DPAB2	1.1	>12
DPAB_mix_	0.030	0.089
DPAC1	>11	>11
DPAC2	>11	>11
DPAC_mix_	>11	>11
DPAD1	>12	>12
DPAD2	4.2	>12
DPAD_mix_	0.20	4.4
DPAE1	4.8	>11
DPAE2	4.3	>11
DPAE_mix_	0.13	6.3

### Effects of DPAmix on enzymes involved in neutral lipid synthesis in CHO-K1 cells

In mammals, TG is synthesized by two major pathways: the glycerol-3-phosphate (G3P) pathway (Supplementary Fig. 2a) and the monoacylglycerol (MAG) pathway. In most cell types, including CHO-K1 cells, TG is mainly produced via the G3P pathway, whereas the MAG pathway is highly active in specific cell lines derived from the small intestine^23^. In the G3P pathway, lysophosphatidate (LPA, which is also known as acylglycerol-3-phosphate) is first synthesized from G3P and fatty acyl-CoA by glycerol-3-phosphate acyltransferase (GPAT). Next, phosphatidate (PA) is synthesized from LPA and fatty acyl-CoA by acyl-CoA:acylglycerol-3-phosphate acyltransferase (AGPAT). Then PA is dephosphorylated by phosphatidic acid phosphatase (PAP) to produce diacylglycerol (DG). Finally, TG is synthesized from DG and fatty acyl-CoA by diacylglycerol:acyl-CoA acyltransferase (DGAT). The direct effect of DPA_mix_ on these enzymes in the G3P pathway was investigated using microsomes prepared from CHO-K1 cells as enzyme sources. As shown in Supplementary Fig. 2b, at the highest concentration of 12 μM, DPA_mix_ showed 23% inhibition of GPAT, 15% inhibition of AGPAT, 6.3% inhibition of PAP and 18% inhibition of DGAT, indicating that the target of DPA_mix_ is not an enzyme in the G3P pathway.

The other neutral lipid, CE, is synthesized from free cholesterol and fatty acyl-CoA by SOAT (also known as ACAT). Therefore, the effects of DPA_mix_ on SOAT activity were investigated using microsomes prepared from CHO-K1 cells. As shown in Supplementary Fig. 2b, DPA_mix_ had no effect on SOAT activity, even at 12 μM. Based on these data, DPA_mix_ had no effects on neutral lipid synthesis in CHO-K1 cells.

### Effects of DPAmix on cellular neutral lipid degradation in CHO-K1 cells

**Figure 3:**
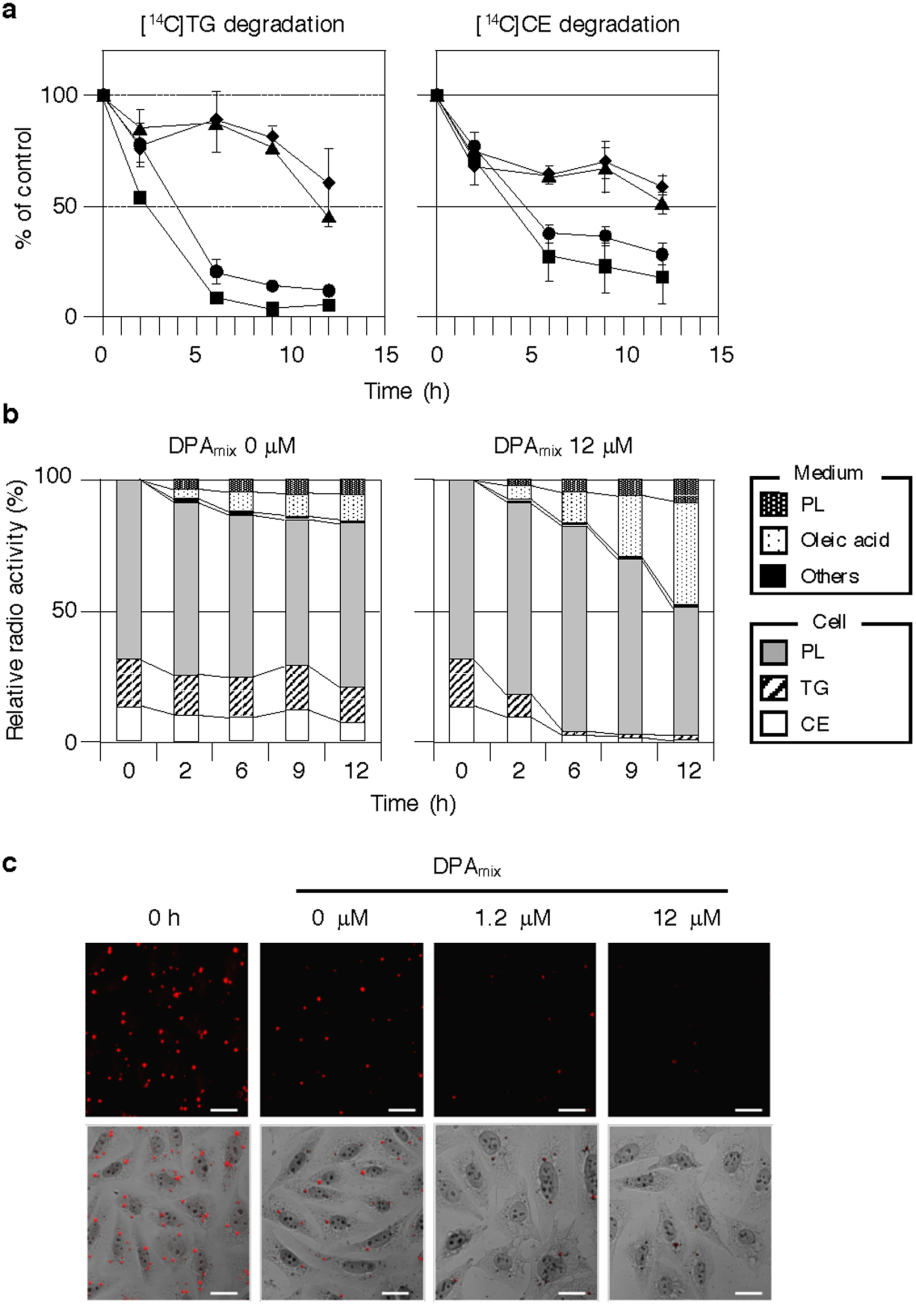
Effects of DPA_mix_ on neutral lipid degradation in CHO-K1 cells. (**a**,**b**) CHO-K1 cells were incubated for 24 h with [^14^C]oleic acid to accumulate [^14^C]lipids. After free [^14^C]oleic acid was removed, and cells were treated with DPA_mix_ for 0–12 h. (**a**) At indicated time, the cells treated with DPA_mix_ 0 (♦), 0.12 (▲), 1.2 (●) and 12 μM (■) were lysed and cellular [^14^C]TG (left) and [^14^C]CE (right) were detected by image analyzer. The results obtained were plotted as % of control (without drugs). Values represent means ± SD (n = 3 ~ 4). (**b**) At indicated time, the cells treated with DPA_mix_ 12 μM and the medium fractions were recovered to analyze the [^14^C]lipids in each fraction using image analyzer. The total amount of [^14^C]lipids in medium and cells is taken as 100% at each time. (**c**) CHO-K1 cells were cultured with 20 μM oleic acid. After the 12 h incubation, the lipid droplet-containing CHO-K1 cells (0 h) were incubated with DPA_mix_ for 6 h, and were stained with oil red O. Bars; 20 μm.

Unexpectedly, DPA_mix_ showed no effect on the enzymes involved in TG synthesis and CE synthesis in CHO-K1 cells. However, the target may be involved in the neutral lipid degradation process. If DPA_mix_ stimulates neutral lipid degradation, neutral lipid synthesis is apparently inhibited. Therefore, additional experiments were performed. CHO-K1 cells were incubated with [^14^C]oleic acid for 24 h to allow [^14^C]TG and [^14^C]CE to accumulate in the cells. After free [^14^C]oleic acid was removed by washing the cells with a bovine serum albumin (BSA)-containing buffer, [^14^C]neutral lipid-loaded cells were incubated with DPA_mix_ (0 to 12 μM). At the indicated times, cells and medium fractions were recovered to analyze the [^14^C]lipids ([^14^C]TG, [^14^C]CE, [^14^C]PL and [^14^C]fatty acids) in each fraction using our established method^24^. As shown in Fig. 3a,b, the amounts of [^14^C]TG, [^14^C]CE and [^14^C]PL in the control cells (0 μM DPA_mix_) gradually decreased, indicating that the degradation of all the lipids proceeded slowly. The half-life times (LT_50_) were calculated to be >12 h. Intriguingly, in the presence of DPA_mix_, decreases in the levels of both [^14^C]TG and [^14^C]CE were observed in the cells that were enhanced in time- and dose-dependent manners, revealing that the degradation of neutral lipids in the cells was enhanced by DPA_mix_. For example, 1.2 μM DPA_mix_ provided an LT_50_s of 3 h for [^14^C]TG and 4 h for [^14^C]CE (Fig. 3a). In the presence of 12 μM DPA_mix_, the cellular [^14^C]TG and [^14^C]CE were clearly degraded within 6 h, and PL was slowly degraded (Fig. 3b). In response to the degradation of cellular [^14^C]neutral lipids, corresponding amounts of [^14^C]oleic acid were recovered from the medium fraction (Fig. 3b).

In parallel with these experiments, neutral lipid-loaded CHO-K1 cells were stained with oil red O and observed under a microscope (Fig. 3c). At time 0, several lipid droplets were observed in the cytosol of cells. After 6 h, the number of lipid droplets in the control cells (without DPA_mix_) was slightly decreased, whereas the number and size of lipid droplets in the DPA_mix_-treated cells were dose-dependently decreased and almost completely disappeared at 12 μM (Fig. 3c). Therefore, the microscopic observation of the effects of DPA_mix_ on neutral lipid-loaded CHO-K1 cells was consistent with the biochemical data for the effects of DPA_mix_ on neutral lipids in CHO-K1 cells. No cytotoxic effects of DPA_mix_ were observed during this experiment (Fig. 3c). Thus, DPA_mix_ promotes the degradation of neutral lipids that accumulated in CHO-K1 cells and the concomitant release of free fatty acids into the medium.

### Effects of DPAmix on autophagy flux

**Figure 4:**
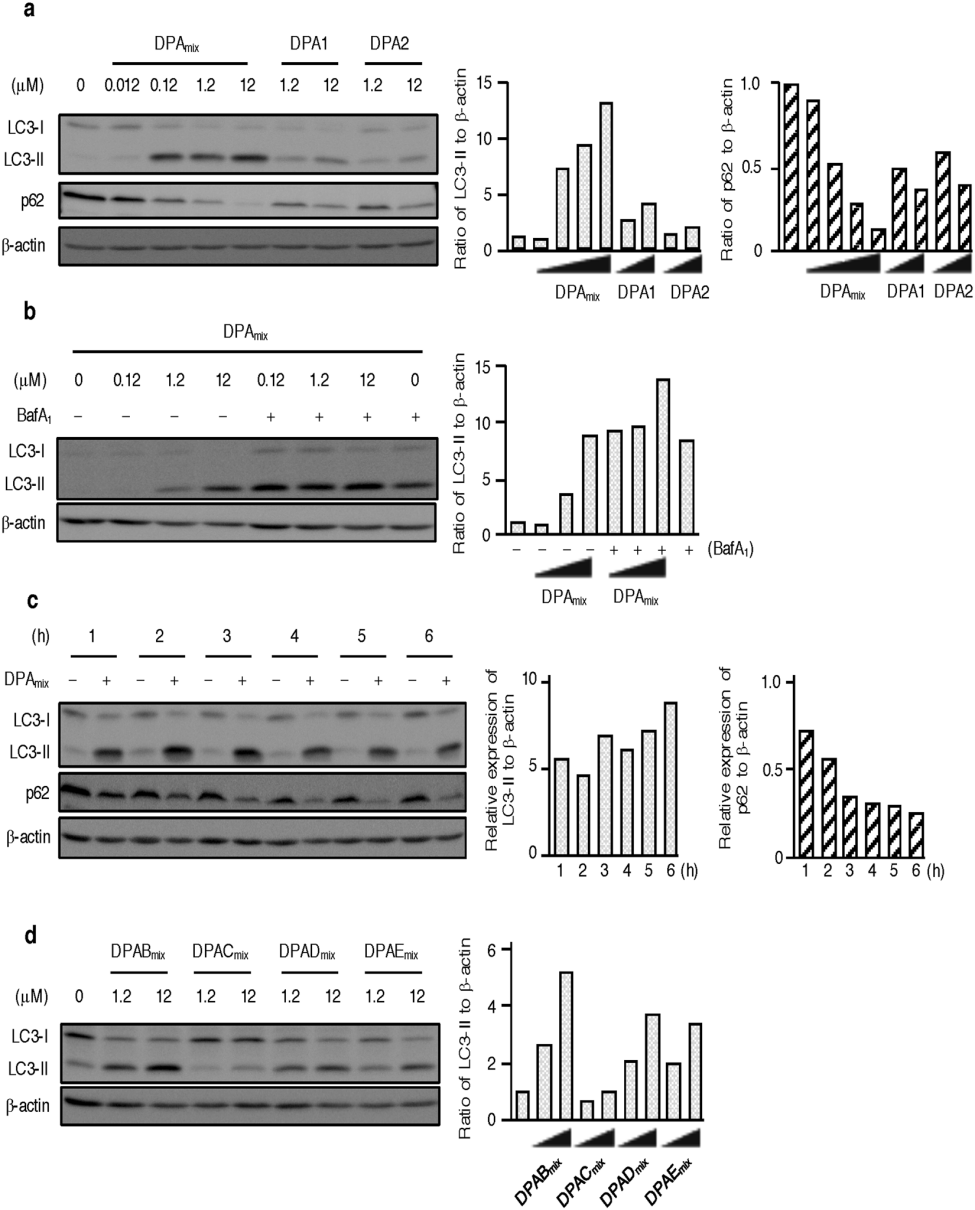
Effects of DPA_mix_ on autophagy flux in CHO-K1 cells. (**a**) After CHO-K1 cells were treated with DPA for 6 h, the cells were lysed and the cell lysates were analyzed by immunoblotting for LC3, p62 and β-actin. (**b**) After CHO-K1 cells were treated with DPA_mix_ (12 μM) for 1–6 h. At indicated time, the cells were lysed and the cell lysates were analyzed by immunoblotting for LC3, p62 and β-actin. (**c**) After CHO-K1 cells were treated with DPAB_mix_, DPAC_mix_, DPAD_mix_ or DPAE_mix_ for 6 h, the cells were lysed and the cell lysates were analyzed by immunoblotting for LC3, p62 and β-actin.

Since DPA_mix_ enhanced neutral lipid degradation in CHO-K1 cells, two catabolic pathways, specifically lipolysis and autophagy (lipophagy), were regarded as a target of DPA_mix_. Lipolysis is the process by which TG is hydrolyzed to glycerol and free fatty acids, and it is highly active in adipocytes^25^. Catecholamines, such as noradrenaline, trigger the lipolysis cascade via the β-adrenergic receptor. However, β-receptors are not expressed in CHO cell lines. In fact, isoproterenol (40 μM)^26^, a β-receptor agonist, had no effects on neutral lipid-loaded CHO-K1 cells (Supplementary Fig. 3). Autophagy is the pathway that degrades intracellular components, including protein aggregates and organelles such as lipid droplets and the mitochondria^27–29^. The process plays a critical role in the maintenance of cellular homeostasis. Upon autophagy induction, microtubule-associated protein light chain 3 (LC3) is recruited to the membrane of autophagosomes and conjugates with phosphatidylethanolamine (PE) to be converted to membrane-bound LC3-II^30^. Therefore, LC3-II is a protein marker of autophagy and is detected with immunoblotting. As shown in Fig. 4a, treatment of CHO-K1 cells with 0.12 μM DPA_mix_ for 6 h markedly increased LC3-II levels, and the levels were constant in cells treated with 0.12–12 μM DPA_mix_. Conversely, approximately the same levels of LC3-I were observed in cells treated with 0 and 0.012 μM DPA_mix_, and the levels decreased in cells treated with 0.12–12 μM DPA_mix_. However, treatment with DPA1 or DPA2 alone only induced a slight increase in LC3-II levels in CHO-K1 cells. The increase in LC3-II levels induced by DPA_mix_ is explained by two possibilities^31^. One possibility is that autophagosome formation is enhanced and autolysosomal degradation occurs. The other possibility is that autolysosomal degradation is inhibited when the docking of autophagosomes with lysosomes is inhibited or lysosomal hydrolase activities are inhibited. To examine the effects of DPA_mix_ on autophagy flux, we performed two assays. First, the level of p62 (known as SQSTM1/sequestosome 1) was measured, because it is selectively incorporated into autophagosomes through direct binding to LC3 and is efficiently degraded by autophagy^32^. As shown in Fig. 4a, the p62 levels in CHO-K1 cells were markedly decreased in a dose-dependent manner after a 6 h treatment with DPA_mix_. Next, we performed an LC3 flux assay^31^. If cells are treated with lysosomotropic compounds such as chloroquine or bafilomycin A_1_ (BafA_1_), which inhibits acidification inside the lysosome or inhibits autophagosome-lysosome fusion, or with lysosomal proteases such as pepstatin A and E-64d, the degradation of LC3-II is blocked, resulting in the accumulation of LC3-II^33^. Accordingly, the differences in the level of LC3-II between samples in the presence or absence of lysosomal inhibitors represent the amount of LC3 that is delivered to lysosomes for degradation. As shown in Fig. 4b, the levels of LC3-II were increased by treatment of BafA_1_ for 6 h. The levels were further increased in co-treatment with DPA_mix_, indicating that autophagic flux was increased by treatment of DPA_mix_. Based on these data, we concluded that DPA_mix_ induces autophagy in CHO-K1 cells. In cells treated with 12 μM DPA_mix_, a marked increase in LC3-II levels was observed within 1 h, indicating that DPA_mix_ is very potent inducer of autophagy (Fig. 4c). Further p62 levels were decreased in a time-dependent manner and completely degraded within 6 h. The LT_50_ of p62 (approximately 2–3 h) in cells treated with 12 μM DPA_mix_ correlated well with the LT_50_ values for [^14^C]TG and [^14^C]CE degradation (Fig. 3a). Furthermore, except for DPAC_mix_, 1:1 mixtures of the atropisomers of heterodimeric DPs (12 μM) also dose-dependently increased the LC3-II levels after 6 h of treatment (Fig. 4d). DPAB_mix_ induced the most potent increase in LC3-II levels, whereas DPAC_mix_ showed almost no induction. These effects of DPs on autophagy markers correlated well with the results for neutral lipid synthesis in CHO-K1 cells (Table 2). Thus, we concluded that DP_mix_ enhanced the degradation of neutral lipids in CHO-K1 cells along with activating the autophagy pathway.

## Discussion

DPs were originally isolated from the culture broth of the fungus *T. pinophilus* FKI-3864 and were determined to be inhibitors of neutral lipid ([^14^C]TG and [^14^C]CE) accumulation from [^14^C]oleic acid in CHO-K1 cells; all DPs consist of (*M*) and (*P*) atropisomers^19–21^ (Fig. 1). DPA2 (*P*) inhibited neutral lipid accumulation, whereas DPA1 (*M*) exhibited almost no inhibition. Interestingly, a 1:1 mixture of DPA1 and DPA2 (DPA_mix_) synergistically inhibited neutral lipid accumulation in CHO-K1 cells (Table 1). In other mammalian cells, such as HeLa S3 cells and HepG2 cells, DPA_mix_ also inhibited [^14^C]TG and [^14^C]CE accumulation rather selectively (Fig. 2). Thus, DPA_mix_ generally inhibits neutral lipid accumulation in most mammalian cell lines. IC_50_ of DPA_mix_ on TG accumulation varied between different cell lines (IC_50_s: 0.054, 2.3 and 9.5 μM for CHO-K1 cells, HeLa S3 cells and HepG2 cells, respectively). This difference might be due to the lipid-producing ability in each cell line. Actually, the radioactivity of [^14^C]TG from [^14^C]oleic acid is almost the same in CHO cells and HeLa S3 cells, but approximately 10 times higher in HepG2 cells. Accordingly, the inhibitory activity of [^14^C]TG accumulation by DPA_mix_ might be weak in HepG2 cells. Other heterodinapinones (heteroDPs) except for DPAC, exerted the same effect as DPA on neutral lipid accumulation in CHO-K1 cells. Specifically, a 1:1 combination of *P* (2 series) and *M* (1 series) atropisomers of each heteroDP synergistically inhibited neutral lipid accumulation in CHO-K1 cells (Table 2). The lower the number of hydroxyl moieties in the alkyl side chains of DPs (3 in DPAB, 4 in DPA, 5 in DPAD and DPAE and 6 in DPAC), the more potent the inhibition of neutral lipid synthesis by (*P*) atropisomers. Based on these results, the number and position of hydroxyl moieties in alkyl side chains of DPs are responsible for the inhibitory effects on neutral lipid accumulation in CHO-K1 cells. In support of this hypothesis, vioxanthin (*P*) with methyl groups as an alkyl side chain lacking a hydroxyl moiety^34,35^ did not inhibit neutral lipid accumulation, even at 18 μM (Supplementary Fig. 4a,b).

Several cases in which a combination of two different molecules elicit synergistic biological activities have been noted. A well-known case is the use of a β-lactam and a β-lactamase inhibitor. The combination potentiates the antibacterial activity of β-lactam against β-lactam-resistant bacteria^36^. Recently, pentamidine, a drug used to treat leishmaniasis, was reported to enhance the antibacterial activity of antibiotics against gram-negative bacteria by disrupting the outer membrane, allowing antibiotics to enter the bacteria and promote cell death^37^. In these two cases, the targets of the two compounds differ. A combination of quinupristin and dalfopristin (w/w, 3:7), both of which are streptogramin antibiotics, show bactericidal activity by inhibiting protein synthesis^38^. The depsipeptide quinupristin binds a nearby site on the 50 S ribosomal subunit to prevent the elongation of polypeptides. The macrolide dalfopristin binds to the peptidyl transferase site in the 50 S ribosomal subunit, which inhibits the earliest process of peptide elongation and results in conformational changes in the 50 S ribosome to enhance the binding of quinupristin to the ribosome. These compounds act on the same target, but at different sites. DP cases are very unusual, and a combination of atropisomers exhibits synergistic inhibition of neutral lipid accumulation in mammalian cells.

We chose DPA as a representative DP and investigated the mechanism of action of DPA_mix_ on lipid metabolism in CHO-K1 cells to obtain a better understanding of this unusual biological activity of DPs. Unexpectedly, DPA_mix_ showed almost no inhibitory effects on enzymes involved in the TG biosynthetic pathway and SOAT involved in the CE biosynthetic pathway, even at 12 μM (Supplementary Fig. 2b). Of course, DPA1 and DPA2 alone did not affect these enzymes. Other possibilities of affecting both TG and CE synthesis are the inhibition of [^14^C]oleic acid uptake into cells or inhibition of acyl-CoA synthetase (ACS), which converts [^14^C]oleic acid to [^14^C]oleoyl-CoA (Supplementary Fig. 2a). However, these possibilities were excluded because the production of [^14^C]PL, which is also produced from [^14^C]oleoyl-CoA, was not blocked by 12 μM DPA_mix_ (Fig. 2). These results strongly suggested that the target of DPA_mix_ is not an enzyme involved in neutral lipid synthesis. In our screening program, intact CHO-K1 cells were incubated with [^14^C]oleic acid for a long time (6 h) in the presence/absence (control) of a sample, and then cellular [^14^C]lipid contents ([^14^C]TG, [^14^C]CE and [^14^C]PL) were measured. When the incubation time (6 h) was reduced (20 min), DPA_mix_ did not inhibit neutral lipid synthesis. Therefore, a long incubation time was necessary for DPA to show inhibitory activity. Another possibility is that DPA was metabolized to active DPA; however, DPA1 and DPA2 are very stable for 6 h under the assay conditions. The last possibility is that DPA_mix_ enhances the degradation of neutral lipids synthesized in the cells. Accordingly, [^14^C]lipid-loaded CHO-K1 cells were prepared, and DPA action was investigated using these cells (Fig. 3). DPA_mix_ time- and dose-dependently enhanced neutral lipid degradation in [^14^C]lipid-loaded CHO-K1 cells. Interestingly, free [^14^C]oleic acid corresponding to the degraded [^14^C]TG and [^14^C]CE in the cells was recovered from the medium, indicating that [^14^C]oleic acid was not degraded via β-oxidation,which probably occurs in peroxisomes to produce ATP and [^14^C]CO_2_. In this neutral lipid degradation experiments, 0.12 μM DPA_mix_ showed no significant change in TG and CE, while IC_50_ values of DPA_mix_ were 0.054 μM for TG and 0.18 μM (CE) in neutral lipid synthesis experiments (Fig. 2 and Table 2). This difference might be due to the difference of amounts of accumulated [^14^C]lipids. In the neutral lipid synthesis experiment, the cells were treated with DPA_mix_ and [^14^C]oleic acid at the same time and thus the [^14^C]TG and [^14^C]CE synthesis from [^14^C]oleic acid and the degradation of [^14^C]TG and CE were started in parallel. In neutral lipid degradation experiment (Fig. 3a), the cells were treated with [^14^C]oleic acid and incubated for 24 h. In this condition, the amounts of cellular [^14^C]TG and [^14^C]CE were approximately ten and five times larger than in the neutral lipid synthesis experiment, respectively. Because of this reason, it is suggested that the inhibitory activity of DPA_mix_ was decreased.

These findings prompted us to investigate the involvement of autophagy to understand the actions of DPA_mix_. As expected, LC3-II, a well-known autophagy biomarker, was clearly observed in CHO-K1 cells treated with 0.12 μM DPA_mix_ for 6 h (Fig. 4a) and in CHO-K1 cells treated with 12 μM DPA_mix_ for 1 h (Fig. 4c), indicating that DPA_mix_ is a very potent inducer of autophagy. DPA1 or DPA2 alone had little ability to induce LC3-II expression (Fig. 4a). Furthermore, p62, another marker of autophagy, was degraded in a dose- (0.012–12 μM DPA_mix_) and time-dependent (0–6 h in the presence of 12 μM DPA_mix_) manner (Figs 4a,c). Notably, the LT_50_ of p62 levels (3–4 h in the presence of 12 μM DPA_mix_) is consistent with the values for [^14^C]TG and [^14^C]CE degradation (2–4 h in the presence of 12 μM DPA_mix_). Thus, the enhancement of neutral lipid degradation and autophagy flus was occurred in parallel. Rapamycin^39–41^ is a well-known inhibitor of mammalian target of rapamycin (mTOR) that negatively regulates autophagy^42^. Unfortunately, rapamycin showed different effects from DPA_mix_ on our CHO-K1 cell assay, as it inhibited [^14^C]CE synthesis (IC_50_: 0.40 μM) and enhanced [^14^C]TG synthesis (Supplementary Fig. 5). The inhibition of mTOR by rapamycin may also affect pathways other than autophagy^43^. Based on this result, the target of DPA_mix_ in the autophagy pathway might not be a component of the mTOR pathway. Furthermore, we have not clearly determined whether DPA1 and DPA2 act on different targets or different sites on the same target and how DPA_mix_ exerts a synergistic effect on the autophagy pathway. In this study, DPA_mix_ enhanced neutral lipid degradation along with induction of autophagy. Further studies are necessary to clarify the mechanism of action of DPA_mix_.

## Materials and Methods

### Materials

All dinapinones were purified from a culture broth of the microorganism *T. pinophilus* FKI-3864 according to established methods^19,21^. Vioxanthin was kindly gifted from Prof. Michael Muller at Institut für Biotechnologie 2, Jülich, Germany. [1-^14^C]Oleic acid (1.85 GBq/mmol) and [^14^C]oleoyl-CoA (1.85 GBq/mmol) were purchased from PerkinElmer (Waltham, MA, U.S.A.). [1-^14^C]Palmitoyl-CoA was purchased from Moravek Biochemicals (Brea, CA, U.S.A.). Phosphatidic acid, L-a-dioleoyl-[2-oleoyl-1-^14^C] (2.04 GBq/mmol) and glycerol, L-[^14^C(U)] 3-phosphate (5.55 GBq/mmol) were purchased from American Radiolabeled Chemicals (St. Louis, MO, U.S.A.). Fetal bovine serum (FBS) was purchased from Biowest (Nuaille, France). Penicillin (10,000 units/ml) and streptomycin (10,000 mg/ml) were purchased from Invitrogen (Carlsbad, CA, U.S.A.). Ham’s F-12 medium, G3P, BSA, 1,2-dioleoyl-*sn*-glycerol, palmitoyl-CoA, Triton X-100, poly L-lysine, oil red O, bafilomycin A_1_, 3-(4,5-dimethylthiazo-2-yl)-2,5-diphenyl-tetrazolium bromide (MTT), isoproterenol and rapamycin were purchased from Sigma-Aldrich (St. Louis, MO, U.S.A.). DMEM (high glucose) medium, diisopropyl fluorophosphates, 2-mercaptoethanol, oleic acid and hematoxylin were purchased from Wako (Osaka, Japan). Nonidet P-40 was purchased from Nacalai Tesque (Kyoto, Japan).

### Cell culture

CHO-K1 cells (a kind gift from Dr. Kentaro Hanada at the National Institute of Infectious Diseases, Tokyo, Japan) and HepG2 cells (purchased from the National Institutes of Biomedical Innovation, Osaka, Japan) were maintained at 37 °C in 5.0% CO_2_ in medium A containing Ham’s F-12 medium supplemented with 10% heat-inactivated FBS, penicillin (100 units/ml) and streptomycin (100 mg/ml) using a previously described method^44^. HeLa S3 cells (a kind gift from Dr. Hideo Iba at the Institute of Medical Science, the University of Tokyo, Tokyo, Japan) were maintained at 37 °C in 5.0% CO_2_ in medium B containing DMEM (high glucose) supplemented with 10% heat-inactivated FBS, penicillin (100 units/ml) and streptomycin (100 mg/ml).

### Assay of neutral lipid synthesis in CHO-K1, HeLa S3 and HepG2 cells

Assays of TG, CE and phospholipid (PL) synthesis in CHO-K1, HeLa S3 and HepG2 cells were performed using our established method^24^. Briefly, CHO-K1 and HepG2 cells (1.25 × 10^5^ cells) were cultured in each well of a 48-well plastic microplate (Corning Co., Corning, NY, U.S.A.) in 250 μl of medium A, and HeLa S3 cells (1.25 × 10^5^ cells) were cultured in each well of a 48-well plastic microplate in 250 μl of medium B. Cells were allowed to recover overnight at 37 °C in 5.0% CO_2_. The assays were conducted when the cells were at least 80% confluent. Following an overnight recovery, a test sample (in 2.5 μl of methanol solution) and [^14^C]oleic acid (1 nmol, 1.85 kBq in 5.0 μl of 10% ethanol/phosphate-buffered saline (PBS)) were added to each well of the culture. After 6 h of incubation at 37 °C in 5.0% CO_2_, cells in each well were washed twice with PBS and lysed with 250 μl of 10 mM Tris-HCl (pH 7.5) containing 0.10% (w/v) sodium dodecyl sulfate; the cellular lipids were extracted using the methods described by Bligh and Dyer^45^. After concentrating the organic solvent, total lipids were separated on a thin-layer chromatography (TLC) plate (silica gel F254, 0.5 mm thick, Merck) in a solvent system of hexane:diethyl ether:acetic acid at a ratio of 70:30:1 (v/v/v), which was analyzed with a bioimaging analyzer (FLA-7000; Fujifilm, Tokyo, Japan) to measure the amount of [^14^C]CE (Rf: 0.77), [^14^C]TG (Rf: 0.55) and [^14^C]PL (Rf: 0.05). IC_50_ values were defined as the drug concentration causing 50% inhibition of the accumulation of each lipid. The concentration of the mixtures was calculated as the average molecular weight.

### Preparation of microsomes from CHO-K1 cells

CHO-K1 cells (2 × 10^8^ cells) were homogenized in 10 ml of Buffer A (100 mM sucrose, 50 mM KCl, 40 mM KH_2_PO_4_ and 30 mM EDTA•4 Na, pH 7.2) supplemented with a protease inhibitor cocktail (Complete mini (Roche)) using a Teflon homogenizer. The microsomal fraction was pelleted by centrifugation at 100,000 × *g* for 1 h at 4 °C, resuspended in the same buffer at a concentration of 5.0 mg/ml protein and stored at −80 °C until use.

### Assay of GPAT and AGPAT activities in microsomes prepared from CHO-K1 cells

GPAT and AGPAT activities were determined as previously described^46^, with a slight modification. Briefly, an assay mixture containing 75 mM Tris-HCl (pH 7.4), 4.0 mM MgCl_2_, 1.0 mg/ml BSA, 150 μM G3P, 28 μ μM palmitoyl-CoA, [^14^C]G3P (1.85 kBq), a sample (added as a 10 µl of methanol solution), and the CHO-K1 microsomal fraction (25 µg of protein) in a total volume of 200 µl was incubated at 25 °C for 20 min. The reaction was started by adding [^14^C]G3P, and stopped by adding 1.2 ml of chloroform:methanol (2:1). Total lipids were extracted using the methods described by Bligh and Dyer. After concentrating the organic solvent, total lipids were separated on a TLC plate in a solvent system of chloroform:methanol:acetic acid:water at a ratio of 65:25:4:2 (v/v/v/v), which was analyzed with FLA-7000 to measure the amount of [^14^C]PA (Rf: 0.39) and [^14^C]LPA (Rf: 0.11).

### Assay of PAP activities in microsomes prepared from CHO-K1 cells

PAP activity was determined as previously described^47^, with a slight modification. Briefly, an assay mixture containing 50 mM Tris-maleate (pH 7.0), 20 mM Triton X-100, 10 mM 2-mercaptoethanol, 2.0 mM MgCl_2_, 0.20 mg/ml BSA and [^14^C]PA (1.85 kBq), a sample (added in 10 µl of methanol solution), and the CHO-K1 microsomal fraction (250 µg of protein) in a total volume of 200 µl were incubated at 30 °C for 20 min. The reaction was started by adding [^14^C]PA and stopped by adding 1.2 ml of chloroform:methanol (2:1). Total lipids were extracted using the methods described by Bligh and Dyer. After concentrating the organic solvent, the total lipids were separated on a TLC plate in a solvent system of diethyl ether:benzene:ethanol:acetic acid at a ratio of 40:50:2:0.2 (v/v/v/v), which was analyzed with FLA-7000 to measure the amount of [^14^C]DG (Rf: 0.66).

### Assay of DGAT activities in microsomes prepared from CHO-K1 cells

DGAT activity was determined as previously described^13^, with a slight modification. Briefly, an assay mixture containing 175 mM Tris-HCl (pH 8.0), 8.0 mM MgCl_2_, 1.0 mg/ml BSA, 2.5 mM diisopropyl fluorophosphate, 150 μM 1,2-dioleolylglycerol, 28 μM palmitoyl-CoA, [^14^C]palmitoyl-CoA (0.74 kBq), a sample (added in 10 µl of methanol solution), and the CHO-K1 microsomal fraction (50 µg of protein) in a total volume of 200 µl were incubated at 23 °C for 15 min. The reaction was started by adding [^14^C]PA and stopped by adding 1.2 ml of chloroform:methanol (2:1). Total lipids were extracted using the methods described by Bligh and Dyer. After concentrating the organic solvent, the total lipids were separated on a TLC plate in a solvent system of hexane:diethyl ether:acetic acid at a ratio of 70:30:1, which was analyzed with FLA-7000 to measure the amount of [^14^C]TG.

### Assay of SOAT activity in microsomes prepared from CHO-K1 cells

SOAT activity was determined using microsomes prepared as described above as the enzyme source^48^. Briefly, an assay mixture containing 2.5 mg/ml BSA in Buffer A, [^14^C]oleoyl-CoA (3.7 kBq), a sample (added in 10 µl of methanol solution), and the CHO-K1 microsomal fraction (50 µg of protein) in a total volume of 200 µl was incubated at 37 °C for 5 min. The reaction was started by adding [^14^C]oleoyl-CoA and stopped by adding 1.2 ml of chloroform:methanol (2:1). Total lipids were extracted using the methods described by Bligh and Dyer. After concentrating the organic solvent, the total lipids were separated on a TLC plate in a solvent system of hexane:diethyl ether:acetic acid at a ratio of 70:30:1, which was analyzed with FLA-7000 to measure the amount of [^14^C]CE.

### Assay of neutral lipid degradation in CHO-K1 cells

CHO-K1 cells (1.25 × 10^5^ cells) were cultured in each well of a 48-well plastic microplate in 250 μl of medium A and allowed to recover overnight at 37 °C in 5.0% CO_2_. Following the overnight recovery, [^14^C]oleic acid (1 nmol, 1.85 kBq in 5.0 μl of 10% ethanol/PBS) was added to each well of the culture and incubated for 24 h to allow [^14^C]lipids to accumulate in cells. Then, the medium was removed, and the cells in each well were washed twice with Buffer B (150 mM NaCl and 50 mM Tris-HCl, pH 7.4) containing 2.0 mg/ml BSA, washed again with Buffer B and subsequently incubated with 250 μl of fresh medium lacking [^14^C]oleic acid in the presence of either DPA_mix_ (2.5 μl of methanol solution) or methanol. After 2, 6, 9 and 12 h of incubation, the medium was collected, the cells were washed and lysed, and the total lipids in both samples were extracted and measured according to methods described above at the indicated time points. The cellular lipid content at each time point was calculated relative to the time point at which [^14^C]oleic acid was removed, which was set to 100%.

### Assay of lipid droplet degradation in CHO-K1 cells using oil red O staining

The assay of lipid droplet degradation in CHO-K1 cells was conducted using a previously described method^49^, with slight modifications. Briefly, CHO-K1 cells (2 × 10^5^ cells) were cultured in 35 mm dishes (Techno Plastic Products AG, Trasadingen, Switzerland) on a glass coverslip coated with poly L-lysine in 2.0 ml of medium A and allowed to recover overnight at 37 °C in 5.0% CO_2_. Following the overnight recovery, 20 μM oleic acid was added to each dish and incubated for 12 h to allow lipid droplets to accumulate in cells. Then, the medium was removed, and the cells in each well were washed twice with Buffer B containing 2.0 mg/ml BSA, washed again with Buffer B and subsequently incubated with 2 ml of fresh medium lacking oleic acid in the presence of either DPA_mix_ (20 μl of methanol solution) or methanol. After 0 and 6 h of incubation, cells were washed with PBS and then fixed by immersion in 10% formalin. Nuclei and intracellular neutral lipid droplets were then stained with hematoxylin and oil red O, respectively. Lipid droplet formation and morphological changes in CHO-K1 cells were examined using a confocal laser-scanning microscope (LSM-510 META, Carl Zeiss, Oberkochen, Germany).

### MTT assay

Assay for cytotoxic activity using CHO cells was carried out by our established method^19^. Briefly, cytotoxicity of a sample to CHO cells was measured by the colorimetric assay on MTT. CHO cells (5.0 × 10^4^ cells in 100 μl) were added to each well of a 96-well microplate. A sample (1.0 μl in MeOH) was added to each well, and the cells were incubated for 12 h at 37 °C. MTT (10 μl of 5.5 mg ml^−1^ stock solution) and a cell lysate solution (90 μl, 40% N, N-dimethylformamide, 20% sodium dodecyl sulfate, 2.0% CH_3_COOH and 0.030% HCl) were added to each well, and the microplate was shaken for 2 h. The optical density (OD) of each well was measured at 540 nm using a microtiter-plate reader (Elx 808; BioTek Instruments, Winooski, VT, USA).

### Western blot analysis of autophagy-related proteins in CHO-K1 cells

CHO-K1 cells (1.25 × 10^5^ cells) were cultured in each well of a 12-well plastic microplate (Corning Co.) in 1.0 ml of medium A and allowed to recover overnight at 37 °C in 5.0% CO_2_. Following the overnight recovery, a test sample was added to each well of the culture. After an incubation at 37 °C in 5.0% CO_2_, cells in each well were washed twice with PBS and detached with trypsin. After centrifugation at 500 x *g* for 10 min, cells were incubated with cold lysis buffer (50 mM Tris-HCl (pH 7.2), 250 mM NaCl, 0.10% Nonidet P-40, 2.0 mM EDTA and 10% glycerol) containing a protease cocktail inhibitor. After 30 min of incubation on ice, lysates were centrifuged at 12,000 x *g* for 10 min at 4 °C, and an equal amount of 2x Laemmli sample buffer containing 2.0% 2-mercaptoethanol was added to each supernatant, which was boiled for 5 min and centrifuged. A portion of the supernatant was separated by SDS-PAGE at a constant current of 20 mA for 1.5 h. Proteins were transferred to polyvinylidene difluoride membranes (PVDF, Immobilon-P, Millipore, USA) for 1 h at 100 V using a Western blot apparatus (MODEL BE-350, BIO CRAFT, Japan). After transfer, the PVDF membrane was blocked in 5.0% skim milk in TBS-T buffer (20 mM Tris-HCl (pH 7.4), 100 mM NaCl and 0.10% (v/v) Tween 20) for 1 h at room temperature. The PVDF membrane was washed with TBS-T buffer and then incubated with a primary antibody (anti-LC3, anti-p62, or β-actin at a 1:2500, 1:1000 or 1:10000 dilution, respectively) in TBS-T buffer for 1 h at room temperature. The primary antibody was then removed, and the PVDF membrane was washed and incubated with a secondary antibody for 1 h. After removing the secondary antibody, the blot was visualized using ECL Western Blotting Detection Reagents (GE Healthcare, Pittsburgh, USA) and detected using a LAS-4000 mini with Science Lab 2005 software (Fujifilm Co., Japan).

## Electronic supplementary material

Supplementary information
