# Supplementary material for: A Mixture of Atropisomers Enhances Neutral Lipid Degradation in Mammalian Cells with Autophagy Induction
Source: Sci Rep. 2018 Aug 14;8:12099. doi: 10.1038/s41598-018-30679-0 (PMC6092391; doi:10.1038/s41598-018-30679-0)

Supplementary information

A Mixture of Atropisomers Enhances Neutral Lipid Degradation in Mammalian Cells with Autophagy Induction

Keisuke Kobayashi, Satoshi Ohte, Taichi Ohshiro, Narihiro Ugaki and Hiroshi Tomoda*

Graduate School of Pharmaceutical Sciences, Kitasato University, Tokyo 108-8641, Japan

* To whom correspondence should be addressed: Hiroshi Tomoda, Graduate School of Pharmaceutical Sciences, Kitasato University, 5-9-1 Shirokane, Minato-ku, Tokyo 108-8641, Japan, E-mail: tomodah@pharm.kitasato-u.ac.jp

Supplementary Figure 1

Effects of DPA_{mix} on cell viability.

Supplementary Figure 2

Effects of DPA_{mix} on the enzymes of neutral lipid synthesis.

Supplementary Figure 3

Effect of isoproterenol on neutral lipid accumulation in CHO-K1 cells.

Supplementary Figure 4

Structure of vioxanthin and the effect on neutral lipid accumulation in CHO-K1 cells.

Supplementary Figure 5

Effect of rapamycin on neutral lipid accumulation in CHO-K1 cells.

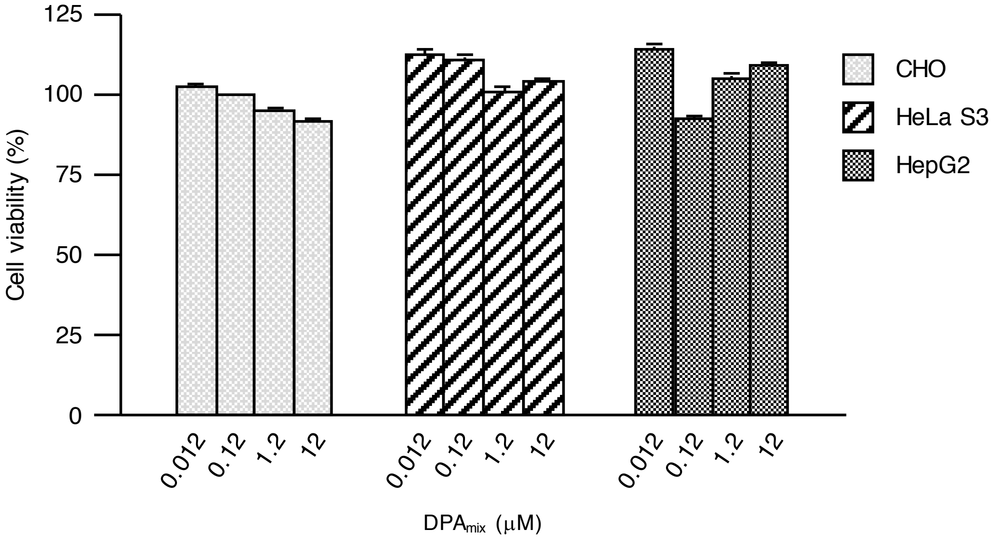

Supplemental Figure 1

Effects of DPA$_{mix}$ on cell viability. After CHO-K1 cells were cultured with DPA$_{mix}$ for 6 h, the cell viability was assayed by MTT. The results obtained were plotted as % of control (without drugs). Values represent means ± SD (n = 3 ~ 4).

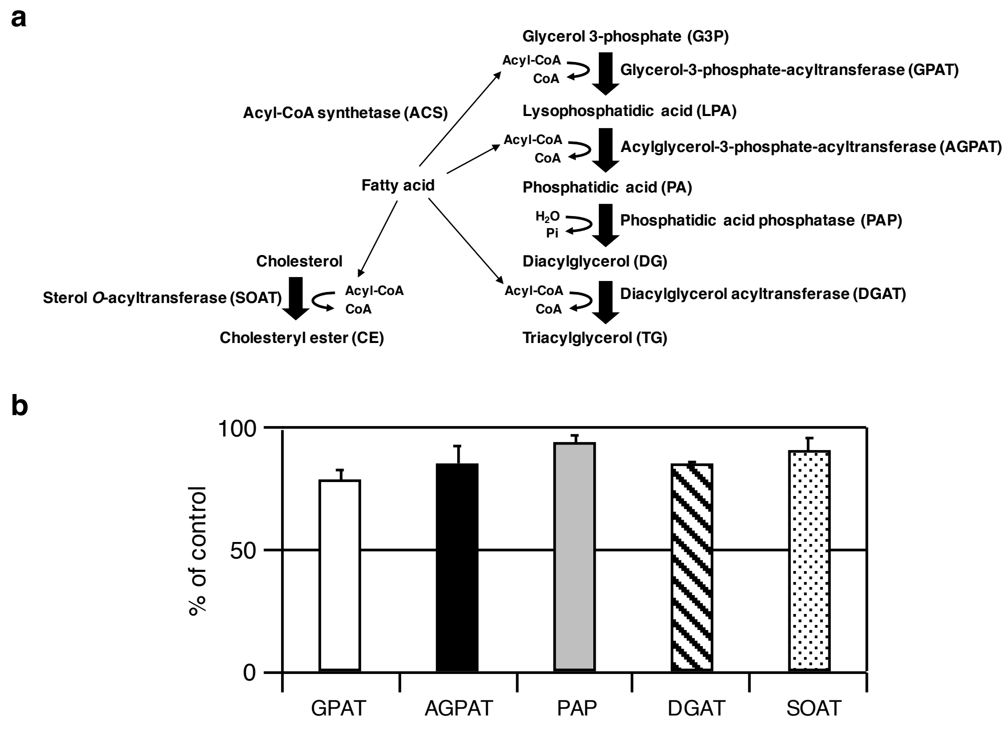

Supplementary Figure 2

Effects of DPA$_{mix}$ on the enzymes of neutral lipid synthesis. (**a**) The biosynthetic pathways of neutral lipids, TG and CE, from fatty acid is described. (**b**) The direct effect of DPA$_{mix}$ (12 μM) on the four enzymes involved in G3P pathway and SOAT was investigated using microsomes prepared from CHO-K1 cells as enzyme sources. The results obtained were plotted as % of control (without drugs). Values represent means ± SD (n = 3 ~ 4).

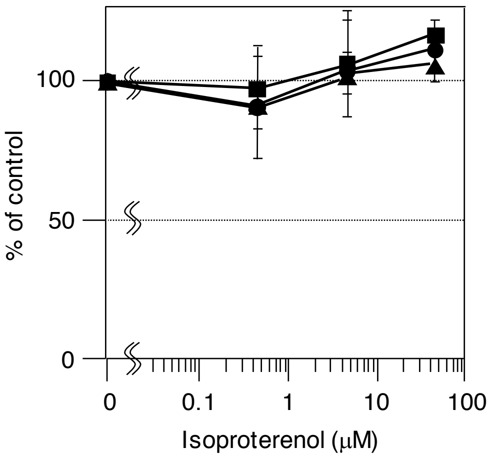

Supplementary Figure 3

Effect of isoproterenol on neutral lipid accumulation in CHO-K1 cells. After CHO-K1 cells were cultured with isoproterenol and [^{14}C]oleic acid for 6 h, the cells were lysed and [^{14}C]TG (■), [^{14}C]CE (●) and [^{14}C]PL (▲) were quantified by an image analyzer.

a **b**

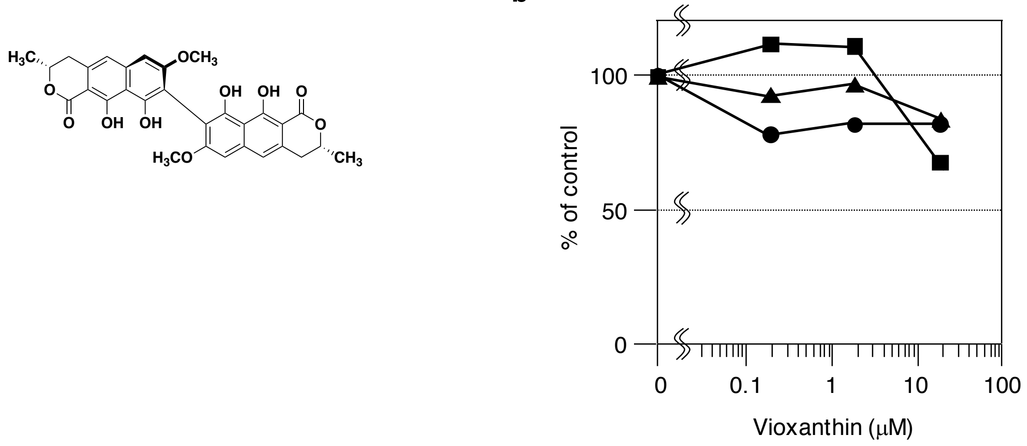

Supplementary Figure 4

Structure of vioxanthin and the effect on neutral lipid accumulation in CHO-K1 cells. (**a**) Structure of vioxanthin. (**b**) After CHO-K1 cells were cultured with vioxanthin and [^{14}C]oleic acid for 6 h, the cells were lysed and [^{14}C]TG (■), [^{14}C]CE (●) and [^{14}C]PL (▲) were quantified by an image analyzer.

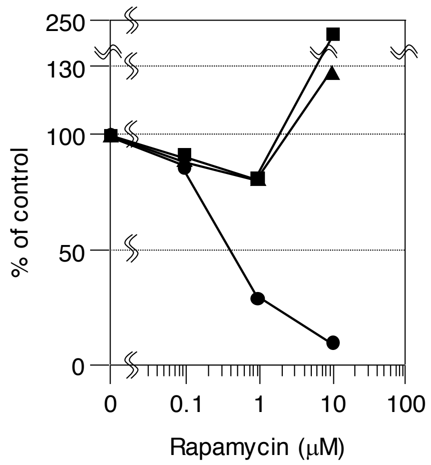

Supplementary Figure 5

Effect of rapamycin on neutral lipid accumulation in CHO-K1 cells. After CHO-K1 cells were cultured with rapamycin and [^{14}C]oleic acid for 6 h, the cells were lysed and [^{14}C]TG (■), [^{14}C]CE (●) and [^{14}C]PL (▲) were quantified by an image analyzer.

Figure 4a

LC3

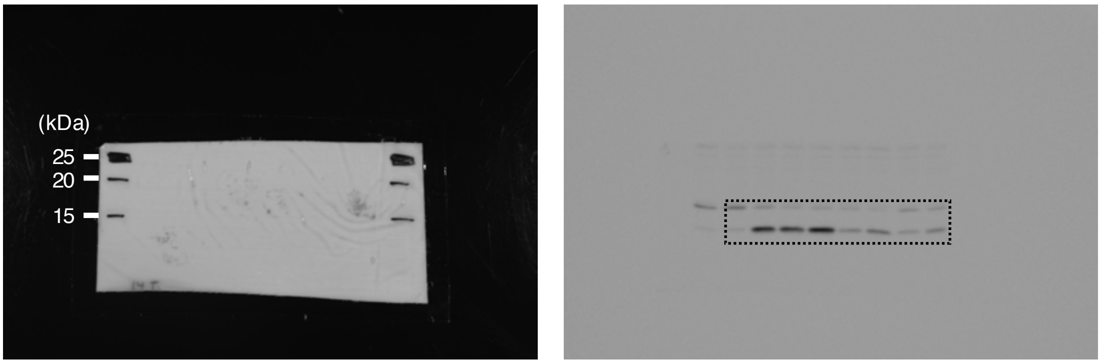

p62

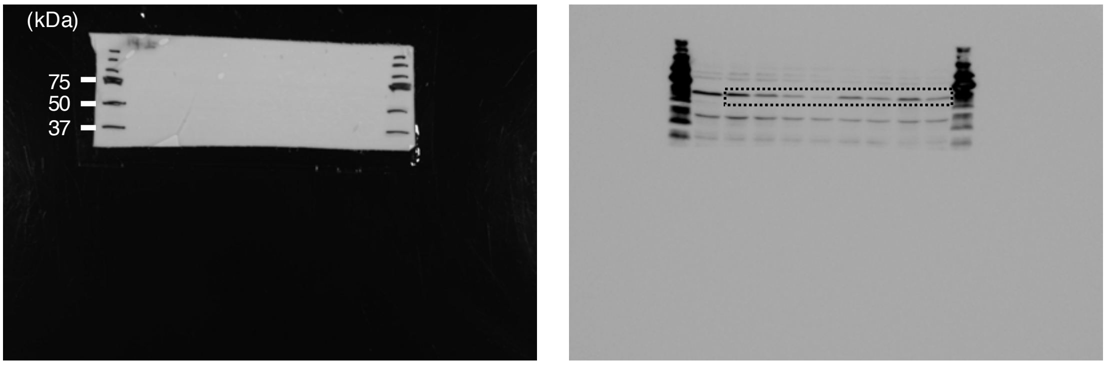

β-actin

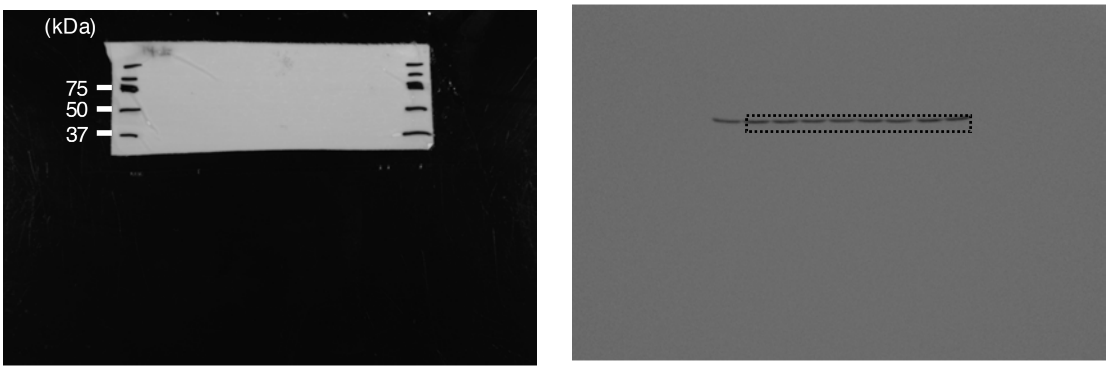

Figure 4b

LC3

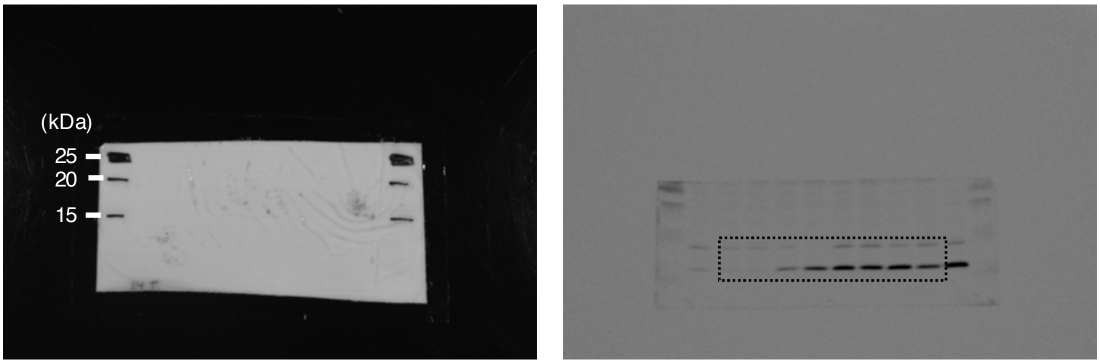

β-actin

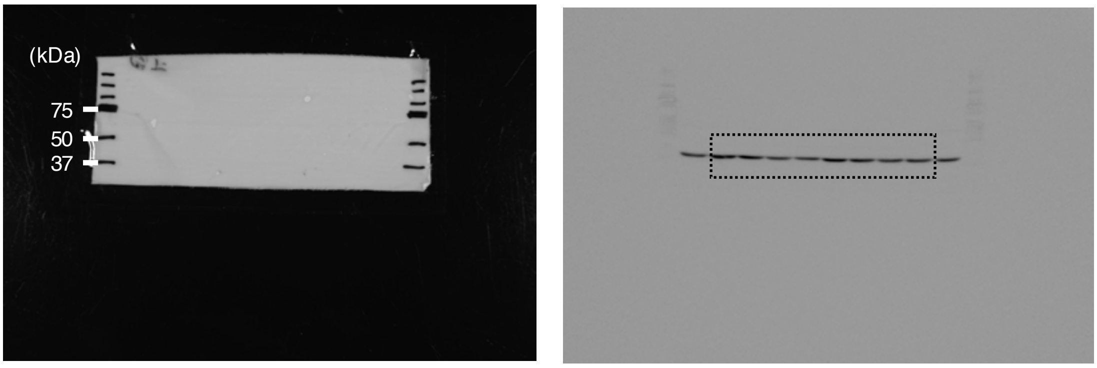

Figure 4c

LC3

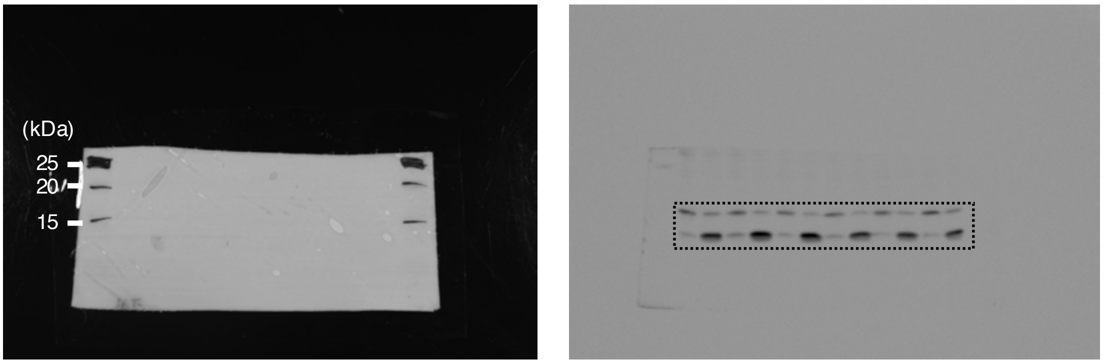

p62

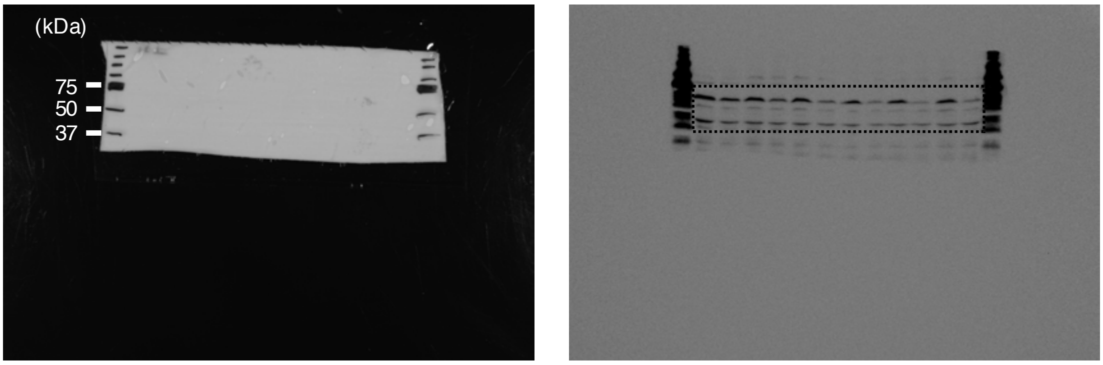

β-actin

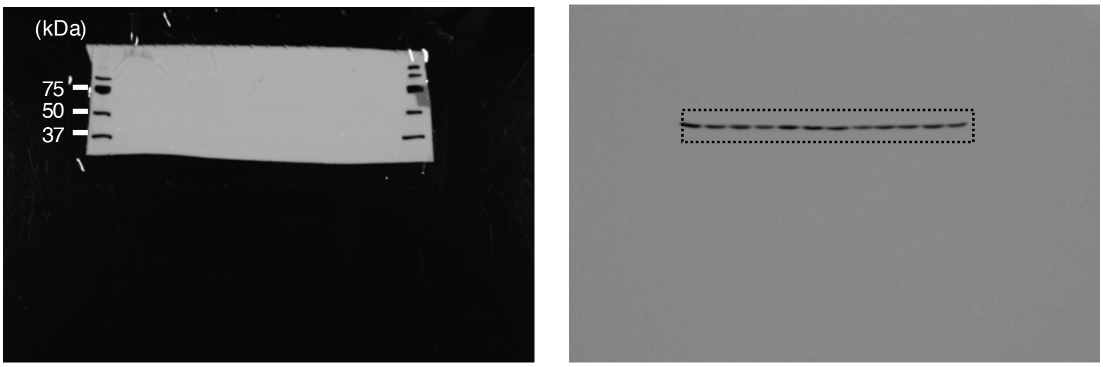

Figure 4d

LC3

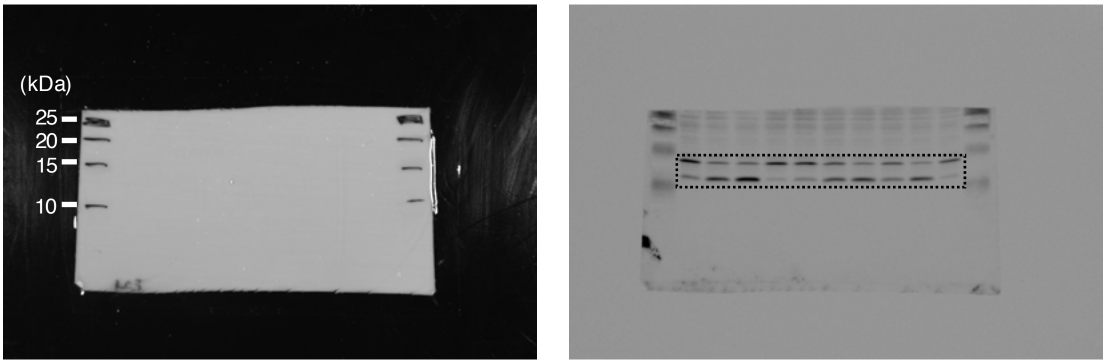

β-actin

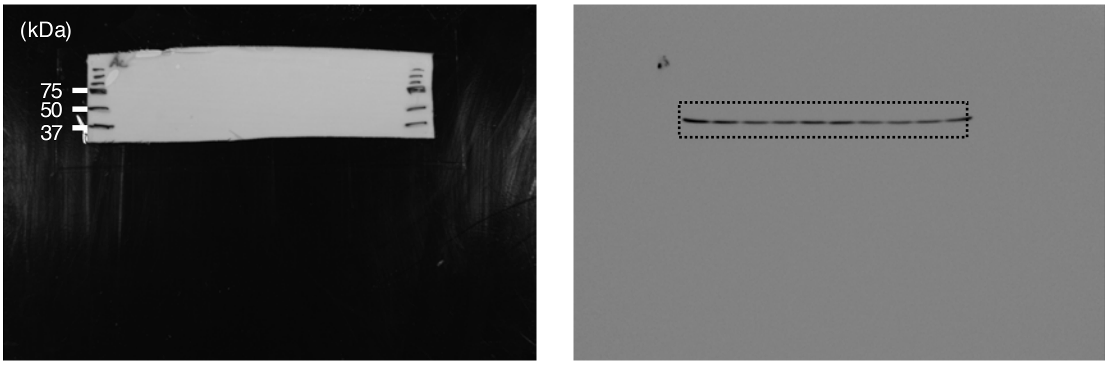

Supplement: Supplementary file 1 — Supplementary information [file 41598_2018_30679_MOESM1_ESM.pdf]
